# Killers on Patrol: Liver Lymphocytes Remain in the Blood Vessels

**DOI:** 10.1371/journal.pbio.0030154

**Published:** 2005-04-05

Protecting the liver from infection presents the immune system with a tough challenge. Because it is the first stop for food absorbed from the gut, the liver is constantly bathed in a rich broth of mostly harmless foreign molecules, which need to be immunologically tolerated. On the other hand, the slow rate of blood flow and high internal surface area makes the liver's many sinuses ideal sites for infections to take hold. One unusual type of immune cell patrolling this complex maze in the liver is the natural killer T (NKT) cell. In this issue, Dan Littman, Mike Dustin, and colleagues shed some light on the behavior of this little-appreciated and important hepatic guardian, showing its unexpected ability to perform immune surveillance entirely within the vasculature. NKT cells crawl along vascular passages in the liver (hepatic sinusoids) at high speeds for a cell moving under its own power, but slow speeds compared to blood flow, and never or rarely venture into the surrounding tissue.

To track the movements of these cells, the authors used a mouse in which the gene for the CXCR6 receptor was replaced with a gene for green fluorescent protein (GFP). In these mice, cells that express CXCR6 also express GFP, and because they fluoresce, they can be visualized and tracked under a microscope. The study showed that cells remained entirely within the blood vessels, crawling along the endothelial lining at approximately 16 microns per minute. Despite directional blood flow, the NKT cells moved and changed directions randomly, and could occasionally be seen migrating past one another in opposite directions within a single vessel. When the authors injected antigen into the blood stream, the cells abruptly stopped, and remained stationary, suggesting that they had fulfilled their first duty to find antigen and were beginning their next: to alert the rest of the immune system. Surprisingly, antigen detection occurred within the blood stream—it is normally thought to occur after lymphocytes leave the blood.[Fig pbio-0030154-g001]

**Figure pbio-0030154-g001:**
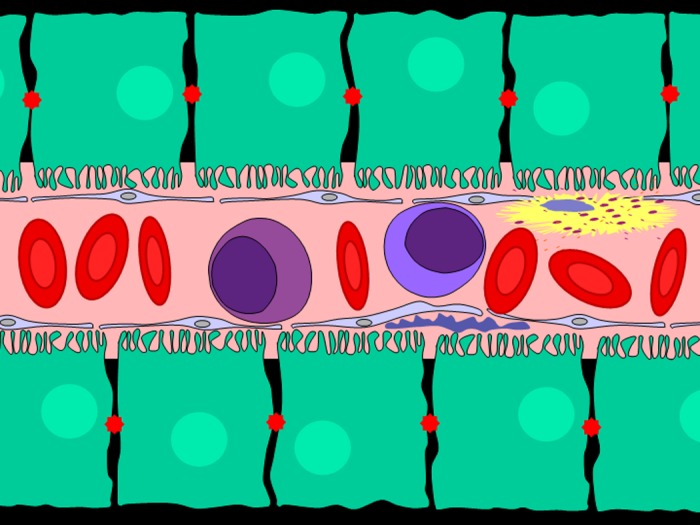
Tracking the movment of glowing T cells in the vasculature

While they make up less than 1% of lymphocytes in other tissues, NKT cells comprise 30% of the lymphocytes in the liver. Mice lacking both copies of the *cxcr6* gene had only one third the normal number of hepatic NKT cells. Though chemokine receptors are typically thought to function by directing cell homing, the authors observed that CXCR6-deficient cells died rapidly in vitro, suggesting that CXCR6 delivers survival signals and thereby controls the number of hepatic NKT cells and liver immune surveillance in general.

While cell numbers were reduced, the movement of the remaining cells in the homozygous-deleted mice was no different than that in the heterozygotes. Because of this, the authors propose that cell motility in wild-type mice is likely to be similar to what they observed in these experiments. Based on the speed and density of the NKT cells, they calculate that each cell can visit a new hepatocyte every two minutes, and that every hepatocyte is surveyed approximately once every 15 minutes. This is in stark contrast to surveillance in the lymph nodes, in which a typical dendritic cell receives 5,000 visits per hour from its resident T cells, but it may make biological sense given that NKT cells have a much more restricted range than conventional T cells.

